# Supplementary figures and images for: Tracking genetic invasions: Genome‐wide single nucleotide polymorphisms reveal the source of pyrethroid‐resistant Aedes aegypti (yellow fever mosquito) incursions at international ports
Source: Evol Appl. 2019 Mar 18;12(6):1136–46. doi: 10.1111/eva.12787 (PMC6597869; doi:10.1111/eva.12787)

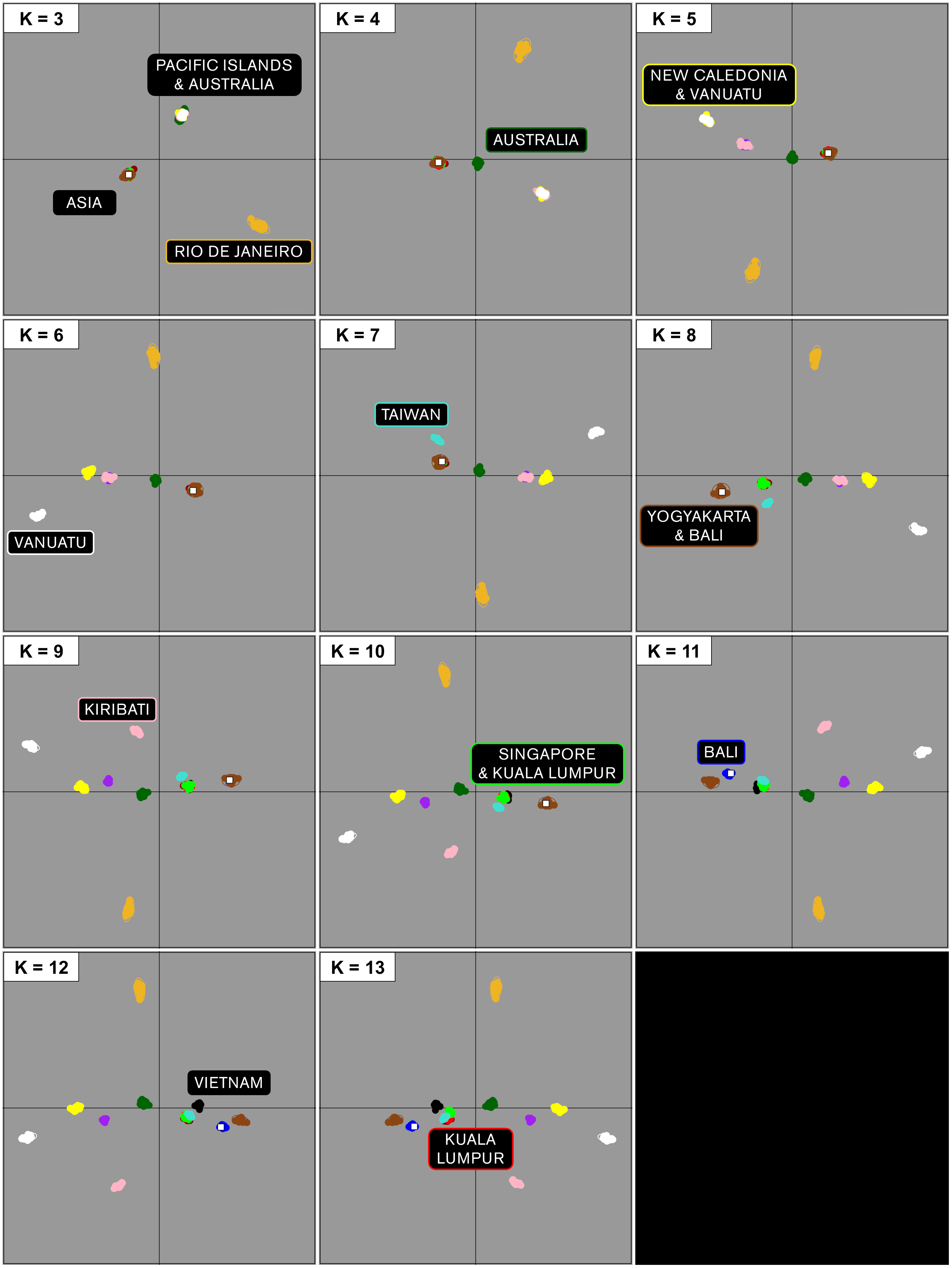

Supplement: Supplementary file 1 [file EVA-12-1136-s001.tif]

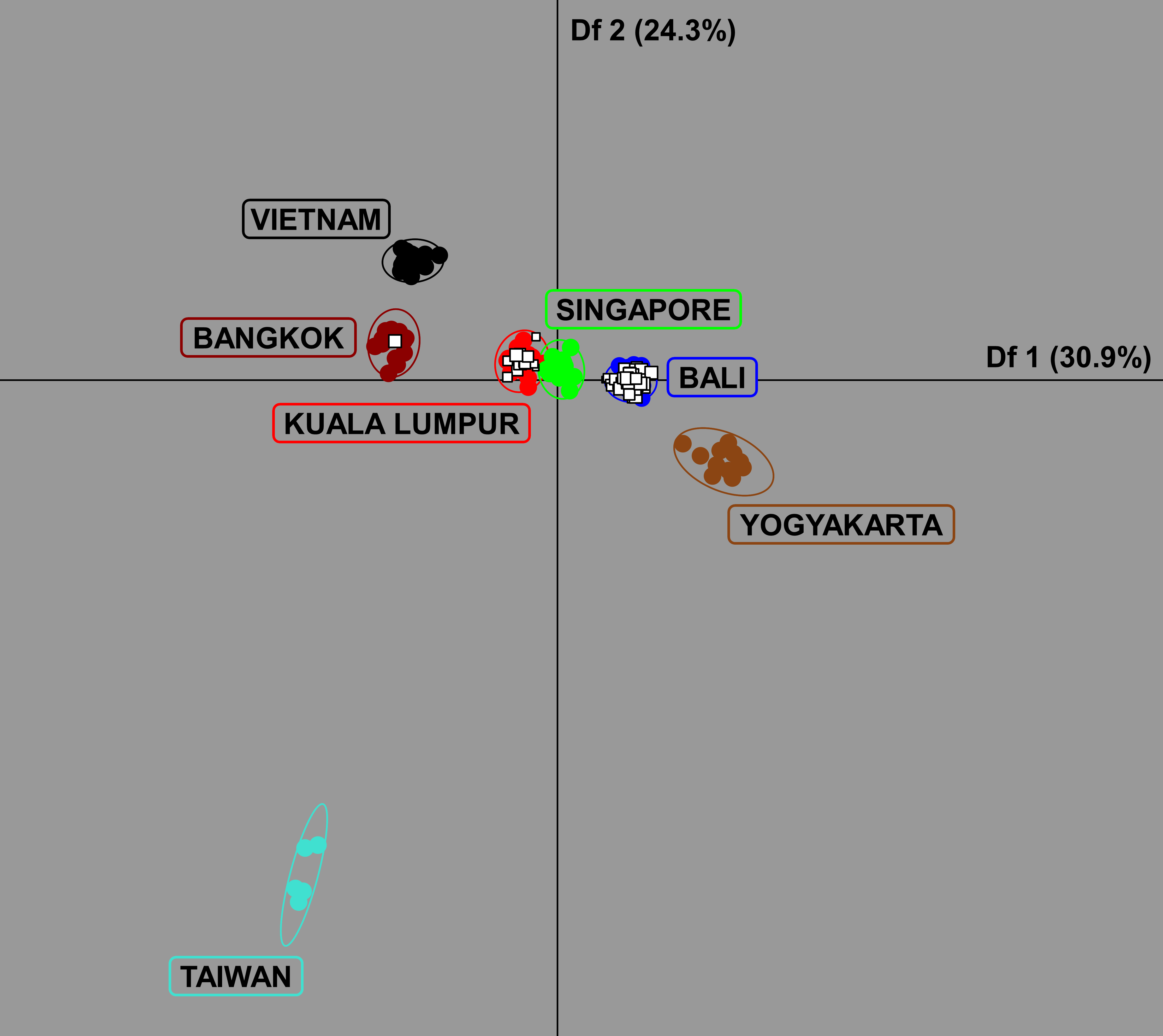

Supplement: Supplementary file 2 [file EVA-12-1136-s002.tiff]
